# Do rats have orgasms?

**DOI:** 10.3402/snp.v6.31883

**Published:** 2016-10-25

**Authors:** James G. Pfaus, Tina Scardochio, Mayte Parada, Christine Gerson, Gonzalo R. Quintana, Genaro A. Coria-Avila

**Affiliations:** 1 Department of Psychology, Center for Studies in Behavioral Neurobiology, Concordia University, Montréal, QC, Canada; 2 Laboratory for the Biopsychosocial Study of Sexuality, Department of Psychology, McGill University, Montréal, QC, Canada; 3 Montréal Neurological Institute, McGill University, Montréal, QC, Canada; 4 Centro de Investigaciones Cerebrales, Universidad Veracruzana, Xalapa, VER, México

**Keywords:** sex, pleasure, animals, male, female, genitosensory, ultrasonic vocalizations, conditioned preference, opioids, dopamine

## Abstract

**Background:**

Although humans experience orgasms with a degree of statistical regularity, they remain among the most enigmatic of sexual responses; difficult to define and even more difficult to study empirically. The question of whether animals experience orgasms is hampered by similar lack of definition and the additional necessity of making inferences from behavioral responses.

**Method:**

Here we define three behavioral criteria, based on dimensions of the subjective experience of human orgasms described by Mah and Binik, to infer orgasm-like responses (OLRs) in other species: 1) physiological criteria that include pelvic floor and anal muscle contractions that stimulate seminal emission and/or ejaculation in the male, or that stimulate uterine and cervical contractions in the female; 2) short-term behavioral changes that reflect immediate awareness of a pleasurable hedonic reward state during copulation; and 3) long-term behavioral changes that depend on the reward state induced by the OLR, including sexual satiety, the strengthening of patterns of sexual arousal and desire in subsequent copulations, and the generation of conditioned place and partner preferences for contextual and partner-related cues associated with the reward state. We then examine whether physiological and behavioral data from observations of male and female rats during copulation, and in sexually-conditioned place- and partner-preference paradigms, are consistent with these criteria.

**Results:**

Both male and female rats display behavioral patterns consistent with OLRs.

**Conclusions:**

The ability to infer OLRs in rats offers new possibilities to study the phenomenon in neurobiological and molecular detail, and to provide both comparative and translational perspectives that would be useful for both basic and clinical research.

Well you tried it just for once found it all right for kicks But now you found out that it's a habit that sticks ‘Orgasm Addict’ by the Buzzcocks (1977)

Orgasms are among the most wonderous and pleasurable events we experience as human beings. The term comes from the Greek term *orgasmos* meaning ‘excitement’ or ‘swelling’ and is synonymous with sexual ‘climax’ or the autonomic apex of copulation or masturbation (Coolen, Allard, Truitt, & McKenna, 2004; McKenna, 1999a). The Roman poet Ovid, in Ars Amatoria (2CE/1855), describes orgasm as a phase which ‘relieves’ or ‘completes’ the lovemaking of both partners (Vol. 2, p. 663). Albert Moll (1908/1912), one of the early German sexologists, described four phases of human sexual response resulting from a ‘tumescence and detumescence drive’ (referring to blood flowing into and out of erectile tissues), positioning orgasms as a ‘voluptuous acme’ or high point in tumescence that yields to a ‘sudden cessation of the voluptuous sensation’ and detumescence (pp. 22–23). Wilhelm Reich (1927/1945) described orgasms as a ‘bioelectric discharge’ of purely sexual tension, breaking away from Freud's (1920/1922) notion of orgasm as a death instinct or ‘Thanatos’ that the life instinct ‘Eros’ always moved its ‘libido’ toward. Kinsey, Pomeroy, and Martin (1948) and Kinsey, Pomeroy, Martin, and Gebhard (1953) noted that orgasms come largely from penile stimulation in men and clitoral stimulation (CLS) in women, although stimulation of other erogenous zones on the body could also sum up to, or in some cases generate, orgasms. Masters and Johnson (1966) reiterated Moll's four stages of human sexual response and identified orgasm at its own phase, the ecstatic period when some or all of the built-up sexual tension is released, followed by a longer-term ‘resolution’ or refractory phase where pelvic muscles and blood flow are relaxed. Despite a plethora of poetry and songs devoted to orgasms and their repercussions, the phenomenology of orgasm in humans is fraught with a multitude of definitions and interpretations, different subjective experiences across individuals, and even different experiences in the same individual across the lifespan. Another problem is that orgasms have occupied the ‘highest’ place in the human sexual response cycle. This drives many people to ‘achieve’ them or somehow ‘give’ them to others, which can diminish the value of sexual sensations experienced during other phases of the response cycle. That said, people's experiences with orgasms, especially first experiences, can be profound and life-changing.

## Genitosensory, motor, and autonomic control of orgasm

**Fig. 1:**
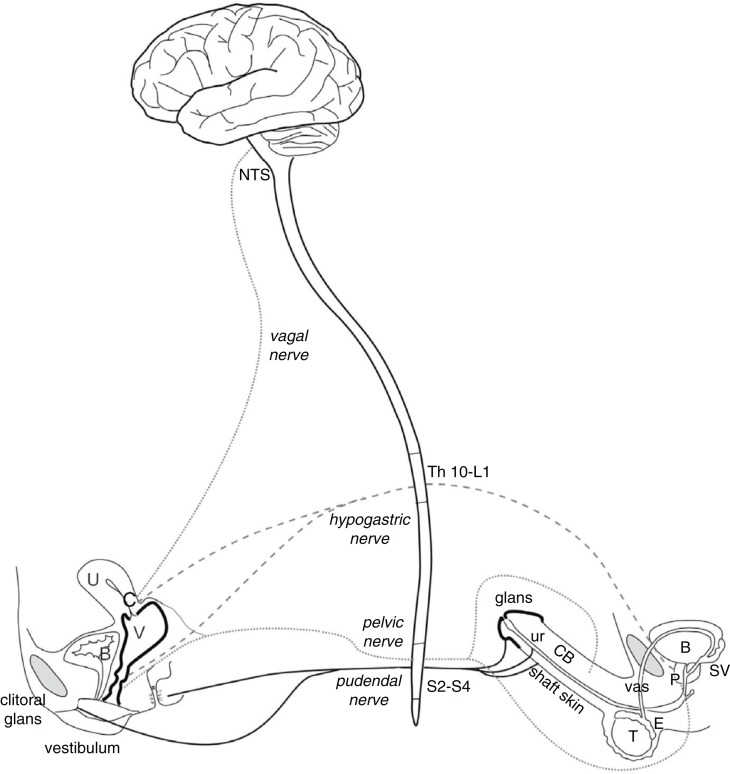
Nerves (pudendal, pelvic, hypogastric, and vagus) that subserve sexual arousal and orgasm in women and men. B: bladder. C: cervix. CB: corpora spongiosum of the bulbocavernosus. E: epididymis. NTS: nucleus of the solitary tract (brainstem). P: prostate. SV: seminal vesicle. T: testis. U: uterus. ur: urethra. V: vagina. vas: vas deferens. Adapted from Georgiadis, et al. (2012).

**Fig. 2:**
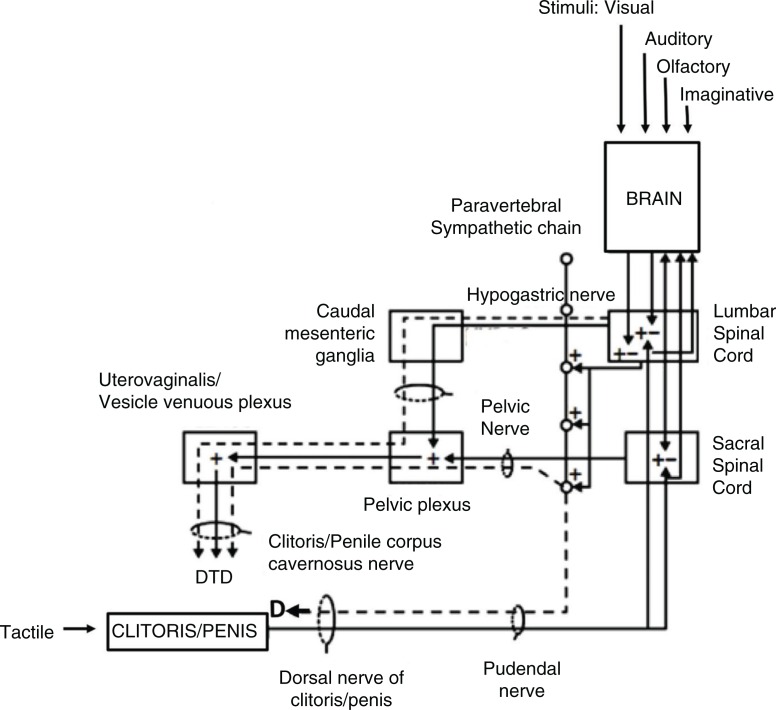
Schematic diagram of the spinal and supraspinal regulation of tumescence (T) and detumescence (D) of the clitoris and penis. Dashed lines are excitatory and solid lines inhibitory for genital blood flow. After De Groat and Steers (1998). LSt neurons that comprise the ejaculation generator in males are located in the lower lumbar cord (see Coolen et al., 2004).

Orgasms are controlled by the autonomic nervous system (ANS) and spinal cord, and ultimately processed in the brain (Komisaruk, Beyer-Flores, & Whipple, 2006). They can be defined and observed objectively as a spinal reflex that results in rhythmic muscle contractions of the pelvic floor and anus, and a urethrogenital reflex often coincident with seminal emission (controlled by the hypogastric nerve) and ejaculation (controlled by the pudendal nerve) in men (Giuliano, 2011; McKenna, 1999b; McKenna, Chung, & McVary, 1991) and contractions of the uterus and cervix in women (Meston, Levin, Sipski, Hull, & Heiman, 2004). Stimulation of the glans and shaft of the erect penis also give rise to pelvic floor muscle contractions as orgasm nears. Stimulation of the vagina and erect clitoris induces pelvic floor muscle contractions (Shafik, 1995; Shafik, El-Sibai, Mostafa, Shafik, & Ahmed, 2005) that appear to increase in duration and intensity as orgasm approaches. The glans penis and external clitoris are homologous structures, and tactile stimulation activates similar sensory nerves (e.g. dorsal clitoral and penile nerves) that course into the pudendal nerve, which enters the spinal cord at the sacral divisions S2–S4 (Fig. 1). The pudendal nerve is a mixed sensory and motor nerve that serves the entire sacral plexus. In addition to the clitoris and penis, the pudendal nerve receives sensory inputs from labia, scrotum, and anus. Its motor division controls contractions of the external urethral and anal sphincters along with the rest of the pelvic floor muscles. The pelvic nerve controls contractions of the internal urethral and anal sphincters and is responsible for erection of the clitoris and penis. Stimulation of the hypogastric nerve activates the sympathetic outflow responsible for orgasm. In turn, the pelvic nerve carries sensory information from the cervix and pelvic floor muscle contractions during orgasm. Input from the cervix is likely also carried by the vagus nerve, which bypasses the spinal cord and sends sensory input directly to the medulla in the brainstem (Komisaruk, Beyer-Flores, & Whipple, 2006; Komisaruk & Whipple, 2005). These nerves thus subserve the genitosensory input to the spinal cord and brain that are processed ultimately as genital swelling, sexual stimulation, and orgasm (Fig. 2). In male rats, a set of spinothalamic neurons in the lower lumbar spinal cord (LSt neurons) has been identified that act as an ejaculation generator, coordinating inhibitory and excitatory outflow from brainstem and hypothalamic nuclei (notably nucleus paragigantocellularis, paraventricular (PVN) nucleus, and medial preoptic area (mPOA)) to control sympathetic, parasympathetic, and motor actions (Coolen et al., 2004; Truitt & Coolen, 2002). It is not known to what extent these neurons play a role in orgasm relative to the control of ejaculation, as ejaculation can occur without corresponding orgasm in men (McKenna, 1999b).

## Neuroanatomical and neurochemical correlates of orgasm

Orgasms produce a similar signature of brain activation in women and men undergoing functional magnetic resonance imaging (fMRI) scans, including deactivations of left ventromedial and orbitofrontal cortices, and activations of anterior cingulate, insula, parietal lobe, hippocampus, amygdala, basal ganglia (especially the putamen), nucleus accumbens (NAc), bed nucleus of the stria terminalis-preoptic area, hypothalamic PVN nucleus, cerebellum (including the anterior lobe of the cerebellar vermis and deep cerebellar nuclei), and lower brainstem (central gray, mesencephalic reticular formation, and nucleus of the solitary tract), with a slight bias for more activation of the central gray in men (Bianchi-Demicheli & Ortigue, 2007; Georgiadis, Kringelbach, & Pfaus, 2012; Georgiadis, Reinders, Paans, Renken, & Kortekaas, 2009; Komisaruk & Whipple, 2005; Stoléru, Fonteille, Cornélis, Joyal, & Moulier, 2012). Using positron emission tomography to analyze regional cerebral blood flow, Huynh, Willemsen, Holstege et al. (2013) found a greater activation of the pituitary following masturbation to orgasm in women relative to men. In another study, similar blood flow to the right ventrolateral pedunculopontine nucleus of the brainstem accompanied ejaculation in men and CLS -induced orgasm in women, with activation patterns corrected for head and facial movements made during ‘faked’ orgasm (Huynh, Willemsen, Lovick, & Holstege, 2013).

Multiple neurotransmitter systems, including endogenous opioids, serotonin, and endocannabinoids, are involved in the subjective feelings of pleasure, satiety, and sedation common to orgasms and their aftermath (Pfaus, 2009). In addition, plasma oxytocin and prolactin levels rise in both men and women during and following orgasm (Krüger et al., 2003, 2006; Murphy, Checkley, Seckl, & Lightman, 1990). The euphoric pleasure of orgasm is likely induced by opioids acting at µ-opioid receptors in different limbic and hypothalamic structures (Ågmo & Paredes, 1988; Pfaus & Gorzalka, 1987; Parada, Sparks, Censi, & Pfaus, 2014). Indeed, heroin addicts describe the rush of euphoria they experience upon injecting heroin in sexual terms, often equating it with orgasm (Chessick, 1960; Pfaus & Gorzalka, 1987). Accordingly, administration of the opioid receptor antagonist naloxone to men blunts the pleasure of orgasm and eliminates the oxytocin release associated with it (Murphy et al., 1990). In addition to the opioid contribution, orgasm depends critically on sympathetic activation, and there appear to be individually defined optimality curves for orgasm across increasing levels of arousal. For example, plasma levels of adrenaline and noradrenaline peak at orgasm and then fall precipitously after, perhaps giving rise to the subjective experience of a ‘release’ of tension and arousal that built up during sexual interaction. Furthermore, drugs or situations that reduce sympathetic tone can delay or abolish orgasm, whereas drugs or situations that increase sympathetic tone can either increase the magnitude or number of orgasms, although such increases must be specific to, or associated with, sexual interaction (Pfaus et al., 2010). However, if stressors are of sufficient magnitude, then the likelihood of becoming sexually aroused is reduced, making sexual behavior and orgasm unlikely to impossible (Barlow, 1986; Both, Everaerd, & Laan, 2003; Magariños & Pfaff, 2016).

## Are orgasms unique to humans?

The study of sexual behavior in animals, mostly rodents and non-human primates, has provided analogies and homologies of human sexual behavior and revealed much about underlying physiological processes that subserve sexual arousal and desire, and to a lesser extent sexual reward and inhibition, that simply could not be obtained from humans (Ågmo, Turi, Ellingsen, & Kaspersen, 2004; Aragona & Wang, 2004; Frohmader, Pitchers, Balfour, & Coolen, 2010; Pfaus, Kippin, & Coria-Avila, 2003; Pfaus et al., 2010, 2012; Wallen, 1995). Accordingly, these models are used widely in sexual and reproductive medicine (Chianese et al., 2011; Giuliano et al., 2010; McMurray, Casey, & Naylor, 2006; Olivier et al., 2006; Pattij, Olivier, & Waldinger, 2005). Mechanisms of erection, ejaculation, and the post-ejaculatory refractory period have been studied in detail in rodents and other species (e.g. Chen, Chan, & Chang, 1999; Giuliano & Rampin, 2004; Levin, 2009; Newman, Reiss, & Northup, 1982). Ejaculation in a variety of species has been viewed as synonymous with sexual ‘climax’ (Fox & Fox, 1971), and indeed the autonomic and neurochemical mechanisms that control erection, seminal emission, and ejaculation are virtually identical in males of a variety of mammalian species, including humans. This begs a question: Can we assume that a male rat or rhesus macaque experiences the pleasure of ejaculation as we experience an orgasm? It may be more accurate to refer to this as an ‘orgasm-like response’ (OLR). Likewise, during copulation, female macaques can display intense tonic/clonic uterine contractions and sudden increases in heart rate coincident with a male's ejaculation (Goldfoot, Westerborg-van Loon, Groeneveld, & Slob, 1980; Slob, Groeneveld, & van der Werff ten Bosch, 1986; Troisi & Carosi, 1998; Zumpe & Michael, 1968), although this response is not always observed. During this time, some female macaques also open their mouths and tense their facial muscles in what Slob et al. referred to as ‘climax face’. Do female macaques or females of other species experience OLRs in response to sufficient copulatory stimulation? And if so, how could we know?

One advantage of studying orgasms in humans is that people can provide a subjective verbal assessment of their feelings that can be contrasted with physiological correlates. However, this comes with a caution: at best, humans do not have a common way to scale orgasm quality or intensity (despite available validated rating scales, like that of Mah and Binik (2002), which researchers doing objective assessments have not utilized); at worst, they lie. Moreover, it is virtually impossible to study human orgasms in any kind of natural environment without necessarily altering the context. And of course, we cannot study first experiences of orgasm except as Kinsey and colleagues did in the subjective retrospective of their participants.

## Inferring subjective states from objective behaviors

Obviously, we do not know what it feels like to be another animal (Nagel, 1974). In fact, our empathy and intuition take us only so far in inferring the feelings of other people. For example, to someone who is sexually active but has never experienced an orgasm, the tales of those that have multiple orgasms must seem outlandish if not also depressing. However, consistent with Dennett (1991), if an orgasm could be distilled into observable (and testable) components, it would be easier to make quantitative determinations of differences in expression that might reflect lawful differences in qualitative experience. What might those components be? A hint is given from the work of Mah and Binik (2002).

In developing their adjective-based rating scale, Mah and Binik (2002) examined factor loadings and structure among a large set of words used by women and men to describe their subjective experience of orgasm from masturbation and copulation. These terms were then rated by several hundred university-age women and men, and then organized by principal components analysis into 12 factors. Further refinement was done by collapsing factors that reflected essentially the same phenomena into a two-factor model that encompassed sensory and cognitive/affective dimensions of the experience of orgasm. This model accounted for 73–84% of the intersubject variance across all measures. The sensory dimension consisted of terms that described sensations of building, flooding, flushing, shooting, throbbing, and muscle spasms. The cognitive–affective dimension described ecstasy, pleasurable and satisfying sensations, relaxation, and emotional intimacy. These dimensions appear to correspond to three components: (1) physical sensations generated by muscle contractions and sympathetic activation, (2) a cognitive/affective sense of ecstasy and pleasure, and (3) the relaxation/intimacy that corresponds to a longer-term refractory period. If we distill these components further into what might be observed in humans or other animals, we can derive at least three phenomena that could characterize an OLR:

1. Physiological changes, including pelvic floor and anal muscle contractions that stimulate seminal emission and/or ejaculation in the male, or that stimulate uterine and cervical contractions in the female. These contractions should occur in temporal synchrony with sympathetic activation and/or bloodflow from erectile tissues;
2. Short-term behavioral changes that reflect immediate awareness of a pleasurable hedonic reward state. These include measurable vocalization patterns, rejection of further genitosensory stimulation, and/or behavioral relaxation or quiescence, during and/or immediately after the OLR; and
3. Long-term behavioral changes that depend on the reward state induced by the OLR. These include an identifiable state of sexual satiety, the strengthening of patterns of sexual arousal and desire in subsequent copulations, and the generation of conditioned place and partner preferences for contextual and partner-related cues associated with the reward state.

How well these criteria might be applied to rats?

## Copulatory responses in rats

To understand the potential expression of OLRs in a rat model, it is necessary to consider the form and patterning of rat copulatory behavior. Copulation in rats is initiated and controlled by females, who enforce or ‘pace’ the multiple mounts, vaginal intromissions, and ejaculations they receive from males (Madlafousek & Hliňák 1978; McClintock, 1984; Pearce & Nuttall, 1961; Pfaus, Jones, Flanagan-Cato, & Blaustein, 2015). Sexually receptive females actively solicit sexual interaction with males by making a headwise orientation to the male followed by an abrupt runaway. This often entices the male to chase them, after which the female holds a pre-lordosis crouch. Males mount with flank palpation, further increasing the lordosis posture of the female, allowing vaginal penetration as the male intromits with brief pelvic thrusts that typically last less than a second. If vaginal penetration has occurred, the male dismounts abruptly and grooms his erect penis into detumescence. The female typically runs away a short distance, and either hops and darts around the male or makes another full solicitation. Hopping and darting typically entices the male to mount again and is used as a proximal solicitation, especially if the male is not chasing or if the female does not have enough room in a testing environment to make a full solicitation with a longer runaway. Finally, stimulation of the cervix results in an inhibitory state. Indeed, in pacing chambers, females typically begin to fight with males after they receive approximately four ejaculatory series and display decreased sexual solicitations and hops and darts (Coopersmith, Candurra, & Erskine, 1996; Pfaus, Smith, & Coopersmith, 1999). This fighting behavior enforces longer and longer intervals between intromissions. Similarly, in large open fields with attached burrow systems, females typically take themselves out of the copulations after a smaller number of ejaculations compared with small chambers (McClintock, 1984). Collectively, these patterns of behavior reflect an ‘estrous termination’ that is dependent on the amount of vaginocervical stimulation (VCS) received by the female (Pfaus, Smith, Byrne, & Stephens, 2000) and corresponding activation of the pelvic nerve (Lodder & Zeilmaker, 1976; Pfaus, Manitt, & Coopersmith, 2006), which in turn activates a glutamate system in the ventromedial hypothalamus (VMH) that inhibits appetitive sexual behavior (Georgescu, Cyr, & Pfaus, 2012; Georgescu, Sabongui, Del Corpo, Marsan, & Pfaus, 2009). Ejaculation by males results in a post-ejaculatory refractory period, after which males resume copulating with the female toward another ejaculation. This can repeat through approximately 6–10 successive ejaculatory series until the male is ‘sexually exhausted’ (Beach & Jordan, 1956) and cannot engage in full copulatory responses for approximately 3–4 days (Rodriguez-Manzo, 1999; Rodríguez-Manzo & Fernández-Guasti, 1994, 1995).

### Male responses to copulatory stimulation

**Fig. 3:**
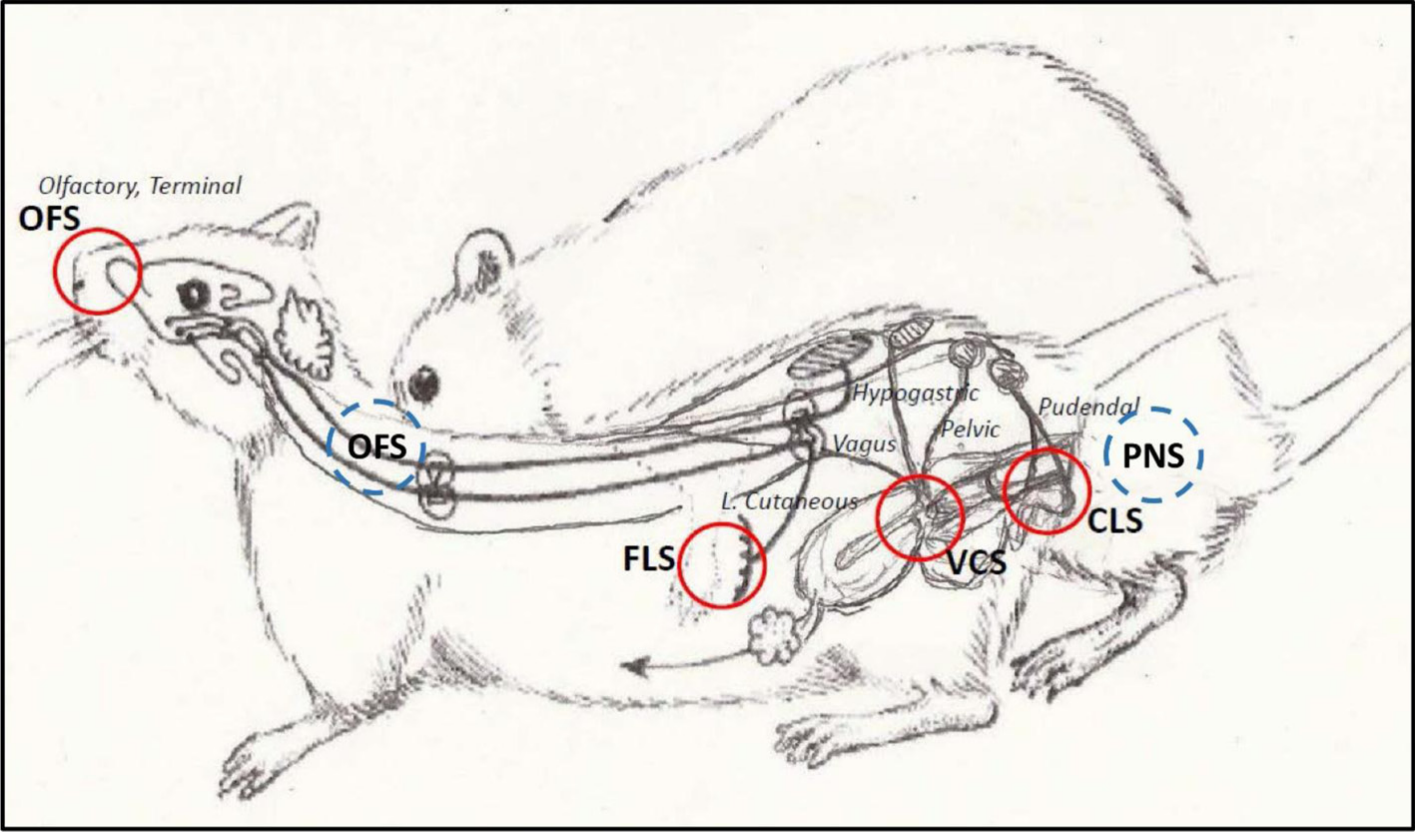
Sensory stimulation of female (red) and male (blue) rats during sexual interaction. OFS: olfactory stimulation. FLS: flank (tactile) stimulation. VCS: vaginocervical stimulation. CLS: clitoral stimulation. PNS: penile stimulation.

In response to female solicitations, male rats mount, intromit, and ejaculate, and males have relatively stable individual patterns of these behaviors (Pattij et al., 2005). In terms of sensory stimulation, males receive both olfactory stimulation from the odors/pheromones of the female along with penile stimulation from intromissions and ejaculations (Fig. 3). Deprivation of sensory stimulation from the penis by topical anesthesia or deafferentation results in males that mount at a higher rate than males given a control treatment but cannot intromit or ejaculate (Gray, Davis, & Dewsbury, 1976; Lodder, 1976). Deprivation of olfactory information, either from destruction of the olfactory epithelium by zinc sulfate, or bulbectomy, abolishes copulatory behavior in males (Carr, Loeb, & Dissinger, 1965; Thor & Flannelly, 1977). However, these disruptions of copulatory behavior do not occur if male rats have sufficient sexual experience (Pfaus et al., 2012), suggesting that sexual experience crystallizes and automates copulatory patterns in males. Males are also aware of their sexual state during copulation. For example, prior to each mount with intromission, males emit a short ultrasonic call in the 50 kHz range (McIntosh, Barfield, & Thomas, 1984; White, Cagiano, Moises, & Barfield, 1990). These precontact vocalizations increase in frequency as males learn to associate the ejaculatory reward state with their pre-ejaculatory copulatory behavior (Bialy, Rydz, & Kaczmarek, 2000; McGinnis & Vakulenko, 2003). These vocalizations are eliminated after castration and restored with exogenous androgen (Harding & Velotta, 2011). Immediately after ejaculation, males emit a long 22 kHz vocalization which corresponds to their period of behavioral quiescence (Burgdorf et al., 2008; McIntosh, Barfield, & Thomas, 1984; White et al., 1990).

### Female responses to copulatory stimulation

Pacing is facilitated for females in large open fields, bilevel chambers (where she can run from level to level), or in unilevel chambers bisected by a Plexiglas partition with holes at the bottom that allow the female – but not the male – to cross from side to side. Females show regular patterns of pacing in these three testing environments. For example, in unilevel chambers with a partition, females display different latencies to return to the male (called ‘contact return latencies’) depending on the preceding copulatory stimulation (short if mounted, longer if intromitted, and longest if she received an ejaculation; Erskine, 1985). These observations bolstered the notion that females enforce an optimal timing between intromissions that leads to optimal timing for the induction of a progestational state (Adler, 1978). If females are tested in small chambers that do not allow efficient pacing, they are more likely to display defensive responses such as rearing or boxing to enforce intervals between mounts, intromissions, and ejaculations. They are also less likely to get pregnant (Frye & Erskine, 1990), suggesting that female rats have a similar ‘vaginal code’ to that first observed in female mice by Diamond (1970).

During copulation, females receive olfactory stimulation from male odors and pheromones, somatosensory stimulation from the flanks (FLS) where males palpate, CLS from pelvic thrusts, and VCS from penile intromissions, which results in the seminal plug after ejaculation (Fig. 3), in addition to the cognitive–affective sense of control given the pacing conditions. Interestingly, VCS alone does not activate LSt neurons in the lower lumbar spinal cord that act in males as the spinal ejaculation generator (Truitt, Shipley, Veening, & Coolen, 2003). However, deprivation of sensory input from the clitoris and cervix through topical anesthesia resulted in shorter contact return latencies in unilevel pacing chambers following intromissions and ejaculations (Meerts, Strnad, & Schairer, 2015). This effect was also observed after transection of the pelvic nerve alone (Meerts & Clark, 2009). In contrast, chronic anesthesia of the external gland of the clitoris alone had the opposite effect in which females had a greater number of exits from and entrances to the male compartment but spent less time with the male (Parada et al., 2013). Females also emit ultrasonic vocalizations in the ‘appetitive’ 50 kHz range during copulation, especially as they approach males (White, Colona, & Barfield, 1991). These calls may be in response to male odor (Matochik, Barfield, & Nyby, 1992) or to the anticipation of rewarding copulatory contact. The calls are displayed reliably in females during proestrus/estrus, are eliminated by ovariectomy, and restored by hormone treatment that restores sexual receptivity (Matochik et al., 1992).

Paced copulation induces more vigorous mounts and intromissions by the male (Erskine, Kornberg, & Cherry, 1989), which result in stronger CLS and VCS from the male's pelvis and penis, respectively, during each mount with intromission and pelvic thrusting.

Together, these data suggest that genitosensory stimulation of the penis or clitoris induce a pleasurable state that males and females are aware of in terms of appetitive calls, and which they attempt to compensate for behaviorally if the stimulation is reduced by pharmacological agents or lesions to specific nerves.

## Effects of repeated pleasurable copulatory stimulation

**Fig. 4:**
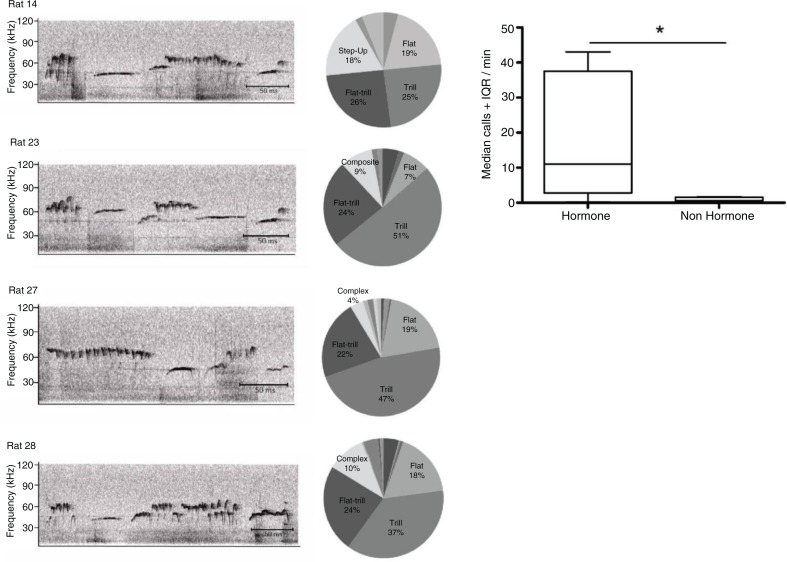
Ultrasonic vocalizations made by female rats in response to distributed CLS. Left side depicts raw calls. Middle depicts the proportion of total calling during CLS taken by flats, trills, flat-trills, step-ups, and compound calls. Right side depicts median calls in females that receive full hormone priming with estradiol benzoate and progesterone versus no hormone (oil vehicle). *P<0.01.

Copulation in both male and female rats also induces a reward state that can be associated by Pavlovian processes with contextual- or partner-related cues to form long-term conditioned place preferences (CPPs) or conditioned partner preferences. CPP is often demonstrated in an apparatus with two distinctive compartments that are connected to either side of a third neutral compartment. During training, the compartments are paired sequentially with different unconditional stimuli, (e.g. one side is paired with the reward state induced by copulation with a sex partner, eating food, or a drug with abuse potential, whereas the other side is paired with either nothing or a control manipulation). In the final test, the animal is placed into the neutral compartment between the two distinctive compartments, with the two doors on either side opened to allow free access to either compartment. CPP is said to have developed if the rat spends significantly more time in the reward-paired compartment than the other compartment. Sexual CPP is observed when male rats are placed into a distinctive compartment of the CPP box after ejaculation, in contrast to being placed into the other compartment after mounts or intromissions alone (Ågmo & Berenfeld, 1990; Everitt, 1990; Hughes, Everitt, & Herbert, 1990; Mehara & Baum, 1990). However, intromissions alone can maintain copulatory CPPs in males that have not yet experienced ejaculation (Hughes et al, 1990; Tenk, Wilson, Zhang, Pitchers, & Coolen, 2009). For females, sexual CPP is achieved by pairing either paced copulation or non-paced copulation with one of two distinct environments (Afonso, Woehrling, & Pfaus, 2006; Paredes & Alonso, 1997; Paredes & Vazquez, 1999). CPP can also be induced by CLS alone (Parada, Chamas, Censi, Coria-Avila, & Pfaus, 2010). We have mimicked the CLS females receive from males by applying it to the clitoris directly with a #4 camelhair paintbrush (Parada et al., 2010; Parada, Abdul-Ahad, Censi, Sparks, & Pfaus, 2011). CLS applied in intervals to mimic its distribution during paced copulation induces a strong CPP relative to continuous CLS or a control condition in which the female's tail is raised but no CLS is applied. Interestingly, the CPP induced by CLS is independent of hormone administration to ovariectomized females (Parada, Vargas, Kyres, Burnside, & Pfaus, 2012). CLS induces immediate 50 kHz vocalizations that follow individual trill-flat patterns (Fig. 4). These patterns are emitted in response to distributed but not continuous stimulation and are maximal in females that are fully sexually motivated and receptive. Thus, like male 50 kHz vocalizations, females appear to be aware of timed CLS and respond to it in a way consistent with the induction of a reward state.

In another group of studies, we have shown that placement of a neutral odor, such as almond or lemon, on a vigorous and receptive sex partner can be paired with the reward state induced by first experiences of ejaculation in males or paced copulation in females. Accordingly, these odors become discrete, partner-related, conditioned stimuli that direct a Pavlovian conditioned partner and mate preference in females (Coria-Avila, Ouimet, Pacheco, Manzo, & Pfaus, 2005; Coria-Avila & Pfaus, 2007) and conditioned ejaculatory preferences in males (Ismail, Gelez, Lachapelle, & Pfaus, 2009; Kippin, Talianakis, Schattmann, Bartholomew, & Pfaus, 1998; Kippin & Pfaus, 2001a, 2001b). Typically, these preferences are formed by paired copulation in bilevel or unilevel pacing chambers and then tested in a large open field with two potential sex partners, one scented and the other unscented. Unpaired controls who received repeated pairing with an unscented partner show a slightly weaker conditioned preference for the unscented partner on the final open-field test, and random-paired controls do not show any conditioning. Strain cues from pigmented versus non-pigmented partners also induce significant partner preferences in both females (Coria-Avila et al., 2006) and males (Ismail, Jones, Graham, Sylvester, & Pfaus, 2011). Interestingly, a female rat given her first paced copulatory experience with one particular male displays conditioned mate-guarding behavior in the presence of that male and a competitor female (Holley et al., 2015; Holley, Shalev, Bellevue, & Pfaus, 2014), a behavior that she does not display if the male is novel or there is no competition for him. These behaviors do not depend on an olfactory cue per se, instead are associated with more ‘pheromonal’ cues (e.g. major histocompatibility complexes) from the familiar male.

The reward state induced by ejaculation or paced copulation is dependent on the release of endogenous opioids in key limbic and hypothalamic brain regions (Paredes, 2014; Pfaus et al., 2012; van Furth, van Emst, & van Ree, 1995; van Furth, Wolterink, & van Ree, 1995). Repeated ejaculations increase whole-brain endorphin content in male rats (Szechtman, Hershkowitz, & Simantov, 1981). Indeed, opioid activation may also mediate the ability of orgasms in humans, and ejaculation in rats, to reduce anxiety and dysphoria (Fernández-Guasti, Roldán-Roldán, & Saldívar, 1989; McCarthy, 1977; Pfaus & Wilkins, 1995; Rodríguez-Manzo, López-Rubalcava, & Fernández-Guasti, 1999). Systemic treatment with the opioid antagonist naloxone, but not dopamine antagonists, during training abolishes the development of CPP and conditioned partner/ejaculatory preferences in both female and male rats (Ågmo & Berenfeld, 1990; Coria-Avila et al., 2008; Ismail, Girard-Bériault, Nakanishi, & Pfaus, 2009; Mehara & Baum, 1990; Paredes & Martinez, 2001). In fact, female rats given their first few sexual experiences under the influence of systemic naloxone lose their desire for sex, as indicated by the loss of solicitations and lordosis, and the induction of defensive responses when injected subsequently with saline (Pfaus et al., 2012). In males, the conditioned ejaculatory preference for the odor is shifted from the familiar scented female to unfamiliar unscented female if naloxone is infused to the mPOA during training, whereas it is abolished altogether if naloxone is infused to the ventral tegmental area (VTA) or NAc during conditioning (Quintana et al., in preparation). Similarly, infusions of naloxone into the mPOA abolish conditioning of CPP in both male (Ågmo & Gomez, 1993) and female (García-Horsman, Ågmo, & Paredes, 2008) rats. Naloxone was also effective in eliminating pacing-related CPP following infusions to the VMH or medial amygdala but not the NAc (García-Horsman et al., 2008). This suggests that the mPOA (and related hypothalamic structures such as the PVN and supraoptic (SON) nuclei) links the reward to particular partner-related cues, whereas the VTA and NAc are involved in the general attending to reward-related cues in males but not necessarily in females. Opioids are known to sensitize dopamine cell activity in the VTA, which results in sensitized release of dopamine in the NAc and elsewhere during reward-seeking behavior (Koob et al., 2014; Kornetsky, 2004; Spanagel, 1995). Opioid actions are also likely responsible for the sensitized activation of oxytocin and vasopressin neurons in the PVN and SON when conditioned rats are in the presence of partner-related cues (Holley et al., 2015).

## The case for OLRs in rats

Both male and female rats display physiological and behavioral changes consistent with the induction of OLRs during sexual interaction. They are clearly aware of the sexual stimuli they receive and behave in ways to maximize the reward received by them. The urethrogenital ejaculatory reflexes of male rats, and the cervicouterine reflexes of female rats, depend on pelvic floor muscle contractions that are experienced at discrete intervals during sex. Responses made to those physiological events include both the induction of short- and long-term inhibitory states for further sexual stimulation, such as refractory periods in males, pacing behaviors by females, along with sexual exhaustion and estrous termination. Longer-term sensitization of contextual- and partner-related cues also occurs that is associated with the induction of a hedonically positive, opioid-induced sexual reward state. This reward state augments the activation of excitatory neurochemical systems, such as dopamine, noradrenaline, oxytocin, and vasopressin (Pfaus, 2009), that drive sexual arousal, CPP, and partner/mate preference in the appropriate circumstances. Thus, female and male rats meet the criteria set forth for OLRs, and they do so with a rich repertoire of behaviors to study beyond copulatory responses alone. Although we can never know if rats experience OLRs the same way we experience orgasms, it is notable that these cognitively mediated behaviors reflect both evaluation and expectation of sexual pleasure or reward. This places them squarely into the realm of responses studied by human sex researchers as a function of sexual pleasure (Georgiadis et al., 2012). In turn, the ability to define OLRs opens new possibilities to study the effects of sexual reward and non-reward, and the manner in which arousal, sexual disinhibition, and other developmental, situational, or partner-related variables might enhance the experience of sexual pleasure from a comparative and translational perspective.
